# Modeling hepatoblastoma development with human fetal liver organoids reveals YAP1 activation is sufficient for tumorigenesis

**DOI:** 10.1007/s13238-021-00893-0

**Published:** 2021-12-10

**Authors:** Li Yang, Jin Chen, Jianqing Liang, Yufeng Zhang, Qingzhe Wang, Xiaojun Ren, Jinsong Wei, Qianchun Gong, Jiting Zhang, Ning Jiang, Xinhua Lin, Jin Li, Bing Zhao

**Affiliations:** 1 State Key Laboratory of Genetic Engineering, School of Life Sciences, Zhongshan Hospital, Fudan University, Shanghai, 200438 China; 2 bioGenous Biotechnology, Inc., Hangzhou, 311231 China; 3 Obstetrics and Gynecology Hospital of Fudan University, Shanghai Key Laboratory of Female Reproductive Endocrine Related Diseases, Fudan University, Shanghai, 200011 China

**Dear Editor**,

Hepatoblastoma (HB) is a predominant hepatic neoplasm that develops in children from 0 to 4 years of age at the rate of 2.16 per 1,000,000. It originates from abnormal differentiation of hepatocyte precursors (hepatoblasts) during embryogenesis (Sumazin et al., 2017). Approximately 20% of children with HB have metastasis in lung at diagnosis, which indicates poor prognosis (Angelico et al., 2019). While surgery in combination of chemotherapy and/or metastasectomy is the most popular therapy, relapse happens in a significant portion of HB patients (Zhang et al., 2021). Therefore, novel and less aggressive therapies targeting the pathogenesis of HB should be explored to prolong patients’s disease-free survival as well as to improve their quality of life.

The in-depth understanding of the molecular mechanisms for HB progression is the key to develop target therapies. But the development of mechanistic studies for HB is rather slow due to the lack of authentic models. Currently, the only way to establish a HB model is to co-overexpress constitutively the constitutively active β-catenin (most frequently mutated gene) and its downstream target, Yes-associated protein 1 (Yap1), in adult mouse liver (Sylvester and Colnot, 2014). This model is quite different from the HB because: (1) its onset is in adult, and (2) there might be pathological differences between mouse and human HB.

Taking advantage of organoid technology, we employed a strategy to establish the human HB initiation model with fetal liver organoids. Based on the recently reported growth-factor-maintained fetal liver organoid culture system (Hu et al., 2018; Prior et al., 2019), we cultivated human fetal liver organoids using 8–12 weeks post-coitum (W) human liver cell clusters. The protocol was optimized by replacing the combination of growth factors with a small-molecule cocktail at day 7 for long-term expansion (Fig. 1A). The human fetal liver organoids retained the tissue-of-origin cell lineages and functions. We observed primary hepatocytes expressing hepatocyte nuclear factor 4 alpha (HNF4A), which can uptake low-density lipoprotein (LDL). We also identified primary bile canaliculi expressing multidrug resistance protein-1 (MDR1), which can transport fluorescein diacetate (Fig. S1). Importantly, the organoids still maintained hepatoblast features including the robust expression of alpha-fetoprotein (AFP) and delta-like 1 homolog (DLK1) (Fig. 1B).

**Figure 1:**
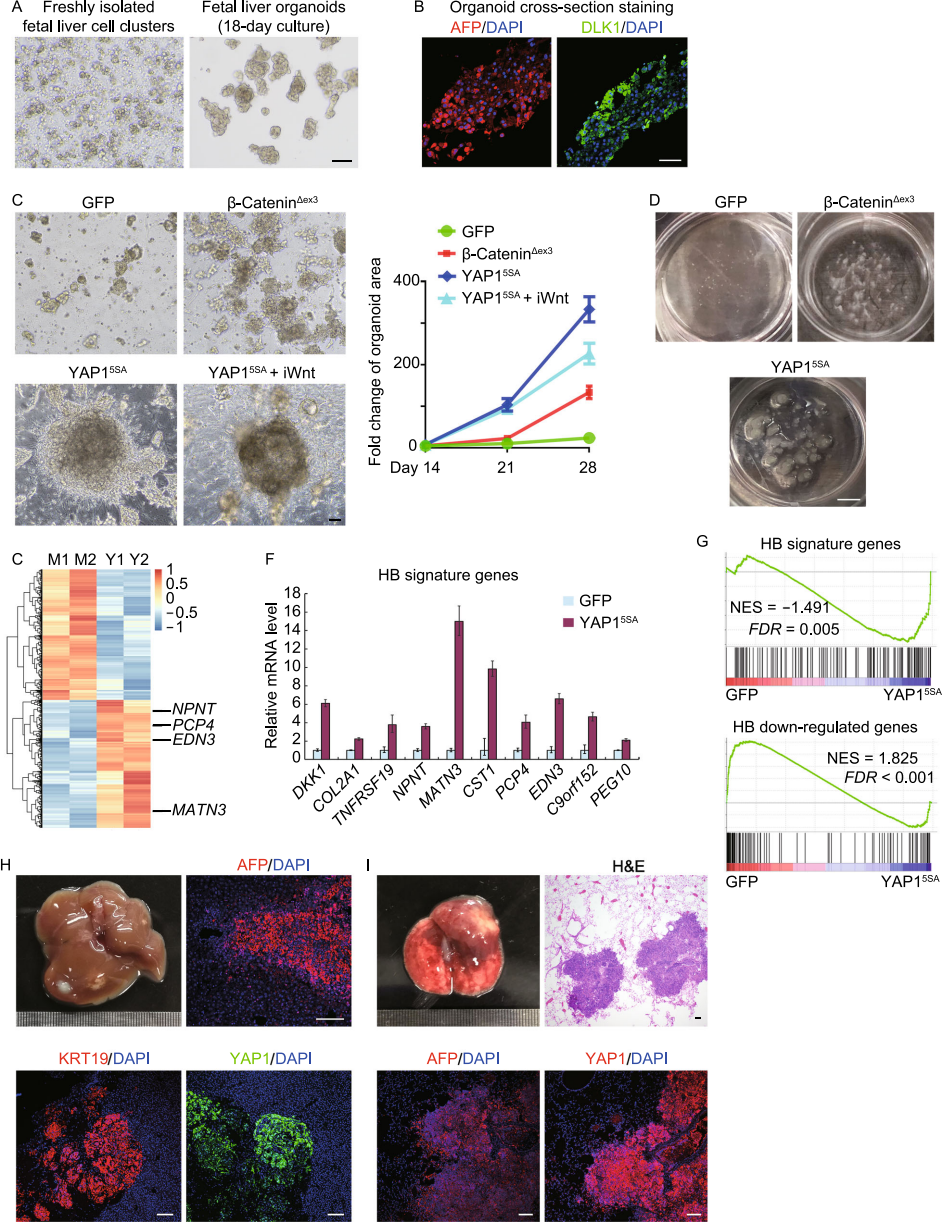
**Modeling hepatoblastoma development with human fetal liver organoids reveals YAP1 activation is sufficient for tumorigenesis.** (A) Brightfield images of freshly isolated human fetal liver cell clusters and 18-day cultured organoids. Scale bar, 200 μm. (B) Immunofluorescence staining for the hepatoblast markers AFP and DLK1. Scale bar, 50 μm. (C) Brightfield images of 21-day cultured fetal liver organoids transfected with lentiviral GFP, β-catenin^Δex3^ or YAP1^5SA^. Growth rate of organoids were quantified. iWnt: Wnt inhibition by 5 μmol/L IWP-2 treatment. Scale bar, 200 μm. (D) Organoids transfected with indicated genes were cultured for 60 days. Scale bar, 250 mm. (E) mRNA expression heatmap of differentially expressed genes for Mock (M1, M2) and YAP1-activated (Y1, Y2) organoids. (F) GFP and YAP1-transfected organoids were harvested to examine the expression of HB signature genes using qRT-PCR. *H3* was used as an internal control. Data were presented as means ± SD (*n* = 3). (G) GSEA enrichment analysis of Mock versus YAP1-activated organoids for HB/normal top 200 genes (top) and HB/normal last 200 genes (bottom). (H and I) YAP1-activated HB organoids were transplanted into the liver capsule of NSG mice. Tumors in liver (H) and metastatic foci in lung (I) were subjected to immunofluorescence staining for AFP, KRT19 and YAP1. Scale bar, 100 μm

We then determined whether the active forms of β-catenin (β-catenin^Δex3^) (Sun et al., 2019) or YAP1 (YAP1^5SA^) (Zhao et al., 2007) can induce HB oncogenesis in fetal liver organoids. Although both Wnt-β-catenin activation and Hippo-YAP activation (Fig. 1C) could promote the proliferation of human fetal liver organoids, only Hippo-YAP activation led to aggressive malignancy (Figs. 1D and S2).

We further dissected the downstream signaling of Hippo-YAP. It has been reported that YAP1 facilitates the nuclear location of β-catenin to activate Wnt-β-catenin signaling (Tao et al., 2014). In consistence, we observed that Wnt-β-catenin activation in YAP1^5SA^-transfected human liver fetal organoids led to significant up-regulation of Wnt-β-catenin target genes (Gene Set Enrichment Analysis (GSEA) in Fig. S3A and qPCR in Fig. S3B). However, suppression of β-catenin transcriptional activity by IWP-2 treatment had no obvious effect on YAP1^5SA^-driven HB progression (Figs. 1C and S4), which demonstrated that Hippo-YAP activation is sufficient for human HB initiation, and that its effects are independent of Wnt-β-catenin signaling. These results suggest that the Hippo-YAP activation, induced by Wnt/β-catenin activation in patients with β-catenin mutations, is the key event for HB initiation.

To validate that the YAP1-activated human fetal liver organoids obtained the key features of HB, we subjected transformed organoids to transcriptome analysis and found 2,176 differentially expressed genes (DEGs) (*P* < 0.05 and fold change ≥ 2) (Fig. 1E). Particularly, Hippo-YAP activation induced the expression of several critical genes for HB oncogenesis, including *DKK1*, *COL2A1*, *THFRSF19*, *NPNT*, *MATN3*, *CST1*, *PCP4*, *EDN3*, *C9orf152*, and *PEG10* (Fig. 1E and qPCR verification in Fig. 1F). GSEA revealed that YAP1-activated organoids significantly increased the expression of “HB signature genes” (Fig. 1G). These data indicated that Hippo-YAP activation transformed fetal liver organoids to HB organoids.

In advance, we tested whether the Hippo-YAP activation induced malignancy by performing orthotopic liver transplantation of fetal liver organoids. In comparison to the control condition, YAP1-activated HB organoids gave rise to xenografts with high expression levels of HB histopathological markers AFP, YAP1, and keratin 19 (KRT19) in 16 out of 22 NOD*-Prkdc*^*scid*^* Il2rg*^*em1*^/*Smoc* (NSG) mice (Fig. 1H). Importantly, 5 of the 16 mice with liver HB exhibited spontaneous lung metastasis thus died (Fig. 1I and survival curve in Fig. S5), which well recapitulated the clinical characteristics of HB (O'Neill et al., 2017). The tumor tissues in lung retained the high expression levels of AFP, YAP1, and NuMA, which confirms that the metastatic foci originated from YAP1-activated human HB organoids (Figs. 1I and S6). Together, these results demonstrated that Hippo-YAP activation in fetal liver organoids well models the HB oncogenesis as well as the malignant progression.

Based on the modeling system, we tried to elucidate the detailed mechanism of how Hippo-YAP activation initiates HB. Several studies have suggested that Hippo-YAP activation induces metabolic reprogramming in hepatocytes. We therefore assessed the metabolic changes of the malignant organoids with LC-MS-based metabolomics. Interestingly, YAP1 activation changed one-carbon metabolism, as S-adenosylmethionine (SAM) was down-regulated while S-adenosyl-L-homocysteine (SAH) was up-regulated in YAP1-activated organoids (Fig. 2A). This result indicated that methyltransferases activity alternation might disturb the balance between SAM and SAH in HB. Moreover, a pan-methyltransferase inhibitor ADOX specifically inhibited the growth of YAP1^5SA^-transfected organoids but not GFP-transfected organoids, suggesting the critical role of methyltransferase in Hippo-YAP-mediated transformation (Fig. 2B). We then scanned a small collection of methyltransferase inhibitors with four DNA methyltransferase inhibitors and 13 protein methyltransferase inhibitors on YAP1-activated organoids. Notably, 4 out of the 6 inhibitors of histone methyltransferase (HMT) G9a (encoded by *EHMT2*), represented by BIX 01294, could specifically attenuate the growth of YAP1-activated organoids (Figs. 2C and S7).

**Figure 2:**
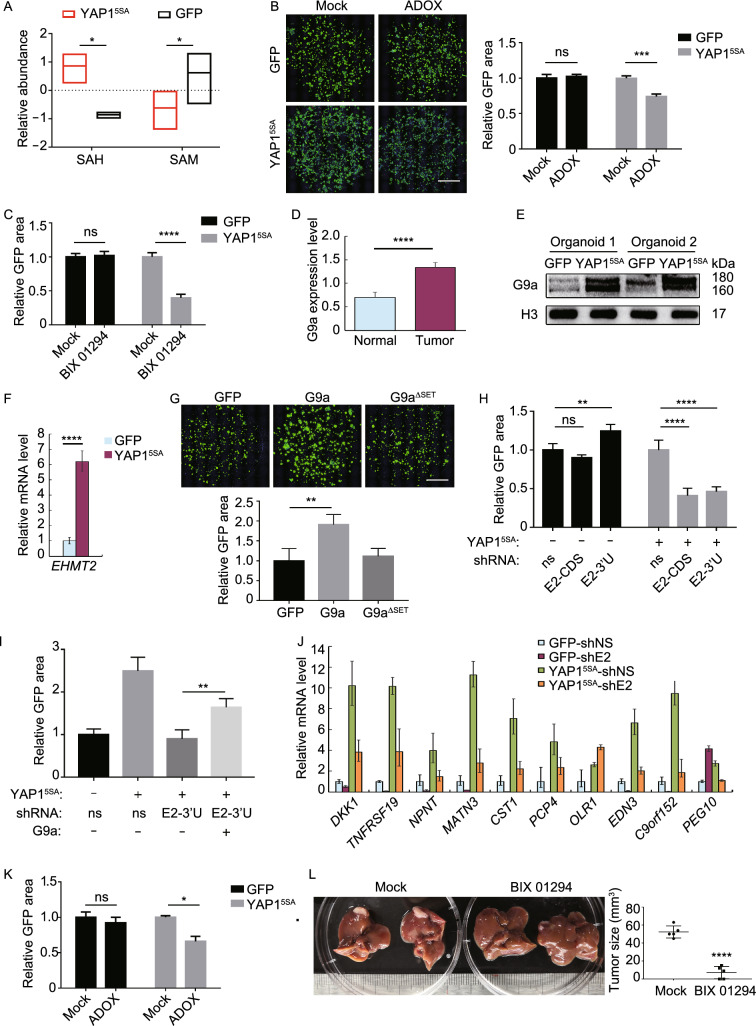
**YAP1 activation initiates hepatoblastoma through up-regulating methyltransferase G9a.** (A) Metabolomic analysis revealed that S-adenosylmethionine (SAM) was down-regulated while S-adenosyl-L-homocysteine (SAH) was up-regulated in YAP1-activated HB organoids. (B) ADOX treatment for 7 days specifically inhibited the growth of YAP1-activated HB organoids. Data were presented as means ± SD (*n* = 4). Scale bar, 1 mm. (C) BIX 01294 treatment for 7 days specifically inhibited the growth of YAP1-activated HB organoids. Data were presented as means ± SD (*n* = 4). (D) The data from HBprem DataBase showed that G9a was up-regulated in HB at protein level when compared to normal tissue. Data were presented as means ± SD (*n* = 5). (E) Western blot determined the expression of G9a in two lines of organoids transfected with GFP or YAP1^5SA^. (F) qRT-PCR analysis of *EHMT2* expression in organoids transfected with GFP or YAP1^5SA^. *H3* was used as an internal control. Data were presented as means ± SD (*n* = 3). (G) Wild-type G9a but not its enzyme-dead mutant ($\Delta$ SET) drove HB oncogenesis. Data were presented as means ± SD (*n* = 4). Scale bar, 1 mm. (H) Knockdown of *EHMT2* by shRNAs inhibited the growth of YAP1-activated HB organoids. E2-CDS: *EHMT2* shRNA targeting CDS; E2-3′U: *EHMT2* shRNA targeting 3′UTR. Data were presented as means ± SD (*n* = 4). (I) Overexpression of G9a restored the growth of YAP1-activated HB organoids transfected with *EHMT2* shRNA targeting 3′UTR. Data were presented as means ± SD (*n* = 4). (J) qRT-PCR analysis revealed that *EHMT2* knockdown down-regulated HB signature genes in YAP1-activated HB organoids. *H3* was used as an internal control. Data were presented as means ± SD (*n* = 3). (K) Human fetal liver organoids transfected with YAP1^5SA^ for 40 days to achieve HB tumorigenesis. These HB organoids were then treated with ADOX for 12 days. Data were presented as means ± SD (*n* = 4). (L) NSG mice were transplanted with YAP1-activated HB organoids to obtain liver tumors. Then, these mice were then received daily treatment of BIX 01294 (5 mg/kg) for 10 days. Data were presented as means ± SD (*n* = 5). * Indicates *P* < 0.05; ** indicates *P* < 0.01; *** indicates *P* < 0.001; **** indicates *P* < 0.0001

*EHMT2*/G9a was up-regulated in both HB (Figs. 2D and S8) and HB organoids (Fig. 2E and 2F). Particularly, overexpression of G9a but not its enzyme-dead mutant drove HB oncogenesis in fetal liver organoids (Fig. 2G). In contrast, knock down of *EHMT2* resulted in growth inhibition of HB organoids (Fig. 2H), which could be rescued by shRNA-resistant *EHMT2* overexpression (Fig. 2I). In addition, *EHMT2* knockdown suppressed HB signature genes in YAP1-activated organoids (Fig. 2J).

Finally, we explored the potential therapeutic effects of methyltransferase inhibitors on HB. We subjected YAP1-transformed HB organoids to the pan-methyltransferase inhibitor ADOX and found that methyltransferase inhibition significantly inhibited the growth of HB organoids at D40 (Fig. 2K). We also determined whether G9a inhibition could target HB *in vivo* by injecting G9a inhibitor BIX 01294 intraperitoneally. As shown in Fig. 2L, G9a inhibition dramatically decreased the size of xenografts one month following the orthotopic transplantation of HB organoids (Fig. 2L), which demonstrated the YAP1-G9a axis is a potential therapeutic target for HB.

In summary, we established a human HB oncogenesis model by manipulating Hippo-YAP signaling in human fetal liver organoids. This model revealed that YAP1 activation is sufficient for human HB tumorigenesis, which overturns the traditional theory that both β-catenin and YAP1 activation are required (Driskill and Pan, 2021; Zhang et al., 2021). Mechanistic studies *ex vivo* and *in vivo* demonstrated that the histone methyltransferase G9a up-regulation is critical for HB oncogenesis. These results highlight the importance of metabolism-epigenetic regulation in HB and suggest that YAP1-G9a could be targeted to develop novel therapeutics.

## Supplementary Information

Below is the link to the electronic supplementary material.

Supplementary file1 (PDF 638 kb)

Supplementary file2 (XLSX 1014 kb)

Supplementary file3 (XLSX 15 kb)

Supplementary file4 (DOCX 1146 kb)
