# Supplementary material for: Modeling hepatoblastoma development with human fetal liver organoids reveals YAP1 activation is sufficient for tumorigenesis
Source: Protein Cell. 2021 Dec 10;13(9):683–8. doi: 10.1007/s13238-021-00893-0 (PMC9233724; doi:10.1007/s13238-021-00893-0)
Supplement: Supplementary file 4 — Supplementary file4 (DOCX 1146 kb) [file 13238_2021_893_MOESM4_ESM.docx]

**Supplementary Information**

**Modeling Hepatoblastoma Development with Human Fetal Liver Organoids Reveals YAP1 Activation is Sufficient for Tumorigenesis**

**Yang et al.**

**Methods**

**Supplementary Figure 1-8**

**Supplementary Table 1-4**

**Methods**

**Human biopsy.** Human fetal liver tissues were obtained and used for research purposes with the informed consent by the patients who requested legally elective abortions at the Obstetrics and Gynecology Hospital of Fudan University. All procedures followed were in accordance with the ethical standards of the Medical Ethical Council of Obstetrics and Gynecology Hospital of Fudan University and with the Helsinki Declaration of 1975, as revised in 2000 (5).

**Mice.** The NOD-Prkdc^scid^ Il2rg^em1^/Smoc (M-NSG) mice were purchased from Shanghai Model Organisms Center, Inc. All animal studies were performed in accordance with the relevant guidelines and under the approval of the Institutional Animal Care and Use Committee of Fudan University.

**Isolation of primary human fetal hepatoblasts.** Fetal liver tissues were kept cold at 4°C in basal-medium until processing. The tissues were minced into pieces of around 2-5 mm^3^ and washed four times using ice-cold wash-medium. Fetal liver pieces were incubated with digestion solution at 37°C for 8 minutes. Ice-cold wash medium was added and digestion mixture was pipetted up and down. After digestion, liver cells were filtered through a 70 μm filter. The material was centrifuged at 60 g for 2 min. The supernatant was discarded and the washing procedure was repeated for three times. Basal-medium: Advanced/DMEMF-12 (Thermo Scientific) supplemented with HEPES, GlutaMax and penicillin/streptomycin. Wash-medium: DMEM (high glucose) supplemented with 1% FBS and 1% penicillin/streptomycin. Digestion solution: collagenase D in EBSS medium at a concentration of 2.5 mg/mL.

**Human fetal liver organoids culture.** Washing supernatant was removed before washing the pellet again with 10 mL of basal medium. Hepatoblasts were mixed with growth factor medium (GF-medium) and Matrigel (ratio=1:3) and then seeded use a volume of 30 μL per 24-well plate. The medium was replaced by fresh GF-medium every three day for up to 7 days. The medium was changed to chemical-defined medium (5C-medium) at day 7. Then the medium was refreshed every 3 days. GF-medium: Basal medium plus B27, 1.25 mM N-acetylcysteine (Sigma), 500 ng/mL R-spondin1 (OrganRegen), 100 ng/mL Noggin (OrganRegen), 50 ng/mL EGF (Invitrogen), 50 ng/mL HGF (OrganRegen), 100 ng/mL FGF10 (OrganRegen), 10 mM Nicotinamide (Sigma), 10 nM gastrin (Sigma), 10 μM Blebbistatin (Selleck), 10 μM Forskolin (Selleck), and 10 μM Rho inhibitor Y-27632 (Calbiochem). 5C-medium: Advanced DMEM/F12 (supplemented with penicillin/streptomycin, GlutaMax, B27 and N-acetylcysteine) plus 1 μM A83-01 (Selleck), 1 μM DAPT (Apexbio), 0.1 μM LDN193189 (Selleck), 20 μM Forskolin (Selleck), and 10 μM Blebbistatin (Selleck). To prepare frozen stocks, organoids were dissociated into single cells, mixed with Organoid Cryopreservation Medium (bioGenous), and frozen following standard procedures.

**Lentiviral vector construction, production, and infection.** β-catenin^Δex3^ and YAP1^5SA^ were generated by mutagenesis. For lentivirus vector construction, β-catenin^Δex3^ and YAP1^5SA^ were cloned into pLVX-P2A-EGFP vector. pLenti6-MK1-EHMT2-V5 was a gift from Bernard Futscher (Addgene plasmid # 31113). MSCVhygro-F-G9a$\Delta$SET was a gift from Kai Ge (Addgene plasmid # 41722). pLKO.1-EHMT2 short hairpin RNA (shRNA) vector was used for establishment of the organoids with *EHMT2* knock down. (Primers for vector construction were listed in Supplementary Table 3). For lentivirus production, triple-plasmid transfection was performed. Briefly, HEK293T cells were transfected with three plasmids (Core plasmid: psPAX2: pMD2.G=7:5:2) using VigoFect (Vigorous) according to the manufacturer’s instructions. 72 h post-transfection, the medium containing virus was centrifuged at 1000 rpm at 4°C for 10 min, then the supernatant was filtered through a 0.45 μm filter. To concentrate the virus, the viral supernatant was centrifuged at 200,000 g at 4°C for 2 h and the virus pellet was dissolved with PBS. For fetal liver organoids at D7, 20 μL lentivirus were co-incubated with organoids in one well of the 24-well plate in 200 μL 5C-medium for two hours at 37°C and shaken every 15 min.

**Histology and immunofluorescence.** For the staining of fetal liver organoids, they were collected in cold PBS, pelleted (60 g, 2 min), and fixed in 4% paraformaldehyde in 4°C for two hours. Following fixation, organoids were washed with PBS, pelleted (60 g, 2 min), and resuspended in 100 μL of warm 2% agarose (in PBS). The cylindrical agarose containing organoids was dehydrated and embedded in paraffin blocks. For the staining of tissues, they were fixed overnight in 4% paraformaldehyde at 4°C, dehydrated, cleared, and embedded in paraffin blocks. For immunofluorescence staining, sections of 5µm were cut and hydrated before staining, and then subjected to antigen retrieval in sodium citrate buffer and permeabilized in PBS supplemented with 0.5% Triton X-100 (PBST). After being blocked with 10% normal goat/horse serum for 1 h at RT, sections were stained with primary antibodies (AFP (proteintech, 14550-1-AP, 1:100), DLK1 (Santa cruz, sc-376755, 1:100), KRT19 (proteintech, 10712-1-AP,1:150), MDR1 (CST, #13978, 1:400), HNF-4α (C-19) (Santa Cruz, sc-6556, 1:200), YAP1 (Santa cruz, sc-15407, 1:200), and NuMA (abcam, ab97585, 1:200)) at 4°C overnight. After washing with PBS, sections were incubated with secondary antibodies. Nuclear staining was performed using mounting medium with DAPI (ASGB-BIO). Specimens were imaged with Olympus FV3000.

**Immunoblotting analysis.** Samples were homogenized in cell lysis buffer (CST, #9803S) supplemented with 1 mM PMSF (CST, #8553S). Cell lysate was centrifuged at 12,000 rpm for 5 minutes at 4°C. Protein supernatant was collected and the concentration was determined using Rapid Gold BCA Protein Assay Kit (Thermo Scientific). Protein was resolved on 8% or 10% polyacrylamide gel and transferred to polyvinylidene fluoride (PVDF) membrane. The membrane was blocked with 5% milk in TBST (0.05% Tween20) for 1 hour at RT, and then incubated with anti-G9a (CST, #3306) and anti-H3 (CST, #4499) at 4°C overnight. The membrane was washed three times in TBST for 10 min and followed by blotting with secondary antibody. Protein expression was visualized with the ECL Buffer (Vazyme) and imaged using the Chemi Doc Imaging System (Bio-Rad).

**Functional analysis of fetal liver organoids.** LDL uptake was evaluated by DiI-Ac-LDL (Biomedical Technologies). To assess secretion function of bile canaliculi, organoids were loaded with 5 μM fluorescein diacetate (Santa Cruz) for 10 min at 37°C and then washed with basal medium. After stained with Hoechst, images were taken using a fluorescence microscope.

**Mouse xenograft studies.** For the orthotopic transplantation, organoids (about 2$\times$10^5^ cells) were collected, washed with 5C-medium, centrifuged to remove all of the supernatant and resuspended in 15 μL of Matrigel. The mixture was injected into the liver capsule of M-NSG mice of aged 5–7 weeks.

**RNA isolation and qRT-PCR.** Total RNA was extracted from organoids using the RNeasy Protect Mini kit (Qiagen) according to the manufacturer’s protocol. One microgram of RNA was reverse transcribed using Goscript^TM^ reverse transcription System (Promega) according to the manufacturer’s protocol. Each PCR was carried out in a volume of 20 μL using SYBR Green Master mix (Promega) in triplicates on the CFX96 Touch System (Bio Rad). Primers used were listed in Supplementary Table 4.

**High-throughput screening.** Fetal liver organoids mixed with Matrigel were seeded on 96-well optical bottom microwell plates (Costar, #3599). Organoids were pretreated with chemical compounds (Adenosine Dialdehyde (ADOX) 10 µM, Epigallocatechin Gallate 10 µM, Decitabine 10 µM, Azacitidine 10 µM, RG108 10 µM, AMI-1 10 µM, Isohomovanillic acid 10 µM, CPI-360 10 µM, GSK503 10 µM, GSK591 10 µM, LLY-283 10 µM, GSK3326595 10 µM, A-366 10 µM, BIX 01294 200 nM, UNC0642 10 µM, UNC0638 10 µM, Chaetocin 0.1 nM, and BRD4770 10 µM) (Selleck) or control (DMSO) for 7 days, or 12 days in 5C-medium at 37°C before screening. Hoechst was applied before screening. One field per well was imaged using the 20× objective and separated into several layers to image 3D organoids on the PerkinElmer Opera Phenix High Content Screening System and the images were extracted using the associated Harmony® Office Software. GFP panel was separated and extracted, and the areas were analyzed using Image J software.

**mRNA sequencing and analysis.** Total RNA was extracted from organoids by using RNeasy Mini Kit (QIAGEN), following the manufacturer’s instructions, and processed with the Ovation® RNA-Seq System V2 Kit (NuGEN) to produce libraries for deep sequencing. Total RNA extraction and library preparation were performed in biosafety level 3 facility according to strict regulations. Libraries were sequenced on an Illumina NovaSeq 6000 platform. After quality control, clean reads were aligned to human reference genome (GRCh38) using HISAT2 (version 2.1.0). The alignments were then passed to StringTie (version 1.3.5) to assemble and quantify the transcripts in each sample. Differentially expressed genes (DEGs) was identified by the R package edgeR (version 3.28.1). Genes were defined as DEGs if they possess the following characteristics: (1) gene expression (FPKM)>1 in any sample, (2) absolute log2 (fold change) ≥2, and (3) *P*-value<0.05. Visualization and hierarchical clustering of log2-transformed FPKM was generated by heatmap (version 1.0.12).

**Organoid hydrophilic metabolite extraction.** Organoid samples were homogenized at -20°C for 15 min. A methanol-water mixtrue (v:v 80:20) was per-chilled at -80°C overnight, and 2 mL of it was added to the sample homogenate. The homogenate was then incubated at -80°C for 20 min and decanted to a 15 mL centrifuge tube. The mixture was centrifuged at 4°C at 4,000 g for 10 min, and then the supernatant was then collected in another 15 mL centrifuge tube. 500 μL of the per-chilled 80% methanol was added to the 15 mL centrifuge tube which contains the sample homogenate, and after 1 min of vertexing, the organoid homogenate was centrifuged at 4°C at 4,000 g for 10 min again. Around 500 μL of supernatant was combined to the 2 mL supernatant in the new 15 mL centrifuge tube. The 2.5 mL supernatant was split to two portions and collected in two 1.5 mL microcentrifuge tubes. The 80% methanol extracted metabolites were dried using SpeedVac (LABCONCO Refrigerated CentriVap Concentrator) and stored at -80°C before the mass spectrometry analysis.

**Targeted metabolomic analysis.** Samples were resuspended in 50 μL of water- acetonitrile mixture (v:v 50:50) and 5 μL of it was injected into a 6500 QTRAP triple-quadrupole mass spectrometer (SCIEX) coupled to HPLC system (Shimadzu). Metabolites were eluted via hydrophilic interaction chromatography (HILIC) by using a 4.6-mm i.d. × 10 cm Amide XBridge column (Waters) with a flow rate of 400 μL/min using buffer A (20 mM ammonium hydroxide/20 mM ammonium acetate (pH 9.2) at a 95:5 ratio of water: acetonitrile) and buffer B (acetonitrile). Gradients were run from 85% buffer B to 42% buffer B at 0-5 min and from 42% buffer B to 0% buffer B at 5-16 min. 0% buffer B was held from 16-24 min. Gradients were run from 0% buffer B to 85% buffer B at 24–25 min, and 85% buffer B was held for 7 min. All the ions were acquired by 306 selected reaction monitoring (SRM) transitions in an alternation of positive and negative modes. ESI voltage was +4,900 V and -4,500 V in positive and negative modes, respectively.

**Gene set enrichment analysis.** Normalized counts of the GFP and YAP1^5SA^ groups were used for gene set enrichment analysis (GSEA), which averaged two biological replicates in each group. The log2-transformed FPKM were used for pre-ranked GSEA, using GSEA software (http://www.broadinstitute.org/gsea/). We generated “HB signature genes” (Supplementary Table 2) and “HB down-regulated genes” gene sets by comparing the transcriptomes between HB and normal tissues (Top 200 and last 200 genes separately of HB/normal, n=5, P value$\leq$0.05) downloaded from HBprem DataBase (http://www.hbpremdb.com/download.jsp). The gene sets database “Wnt signaling” was used (https://www.gsea-msigdb.org/gsea/msigdb/cards/WNT_SIGNALING.html).

**Quantification and statistical analysis.** We employed Student’s *t*-test and two-way ANOVA test to analyze the experimental results. Analyses were conducted on GraphPad Prism 7 statistical software. All values are represented as means±SD. The value of * *P*<0.05, ** *P*<0.01, *** *P*<0.001 was considered significant.

**Supplementary Figure 1**


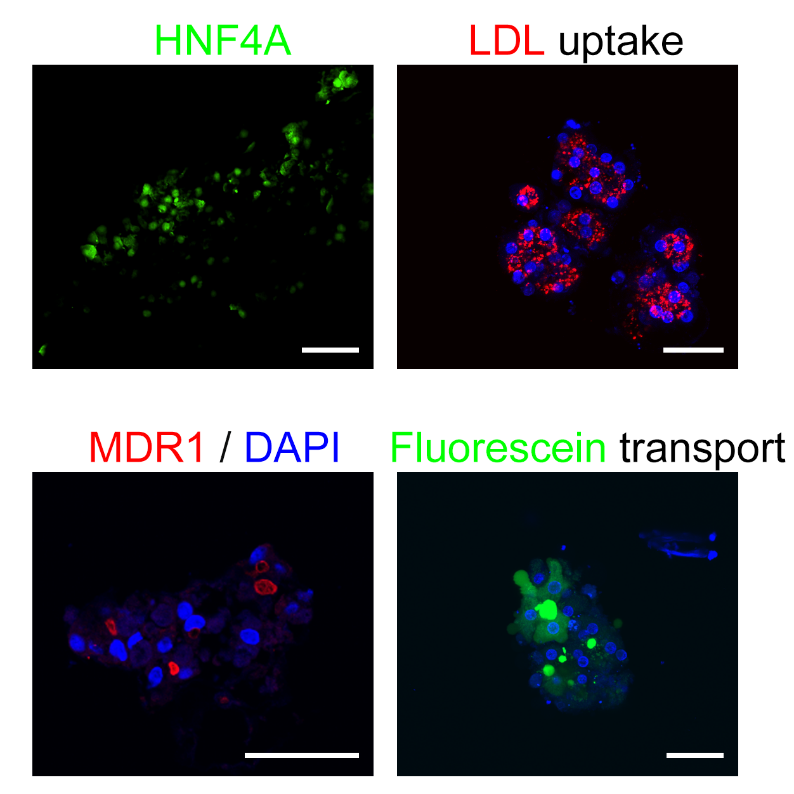


**Supplementary Figure 1 ∣ Fetal liver organoids preserved tissue-specific markers and functions.** Confocal cross-sectioning showed the HNF4A expressed in most cells of human fetal liver organoids. LDL uptake was visualized by Dil-ac-LDL fluorescent staining (red) in cultured organoids. Nuclei were stained with Hoechst (blue). Immunofluorescence staining for the bile canaliculi marker MDR1, which is consistent with the accumulation of fluorescein diacetate (green) in the bile canaliculi structures in cultured organoids visualized by confocal (z stack projection). Nuclei were stained with Hoechst (blue). Scale bar, 50 μm.

**Supplementary Figure 2**


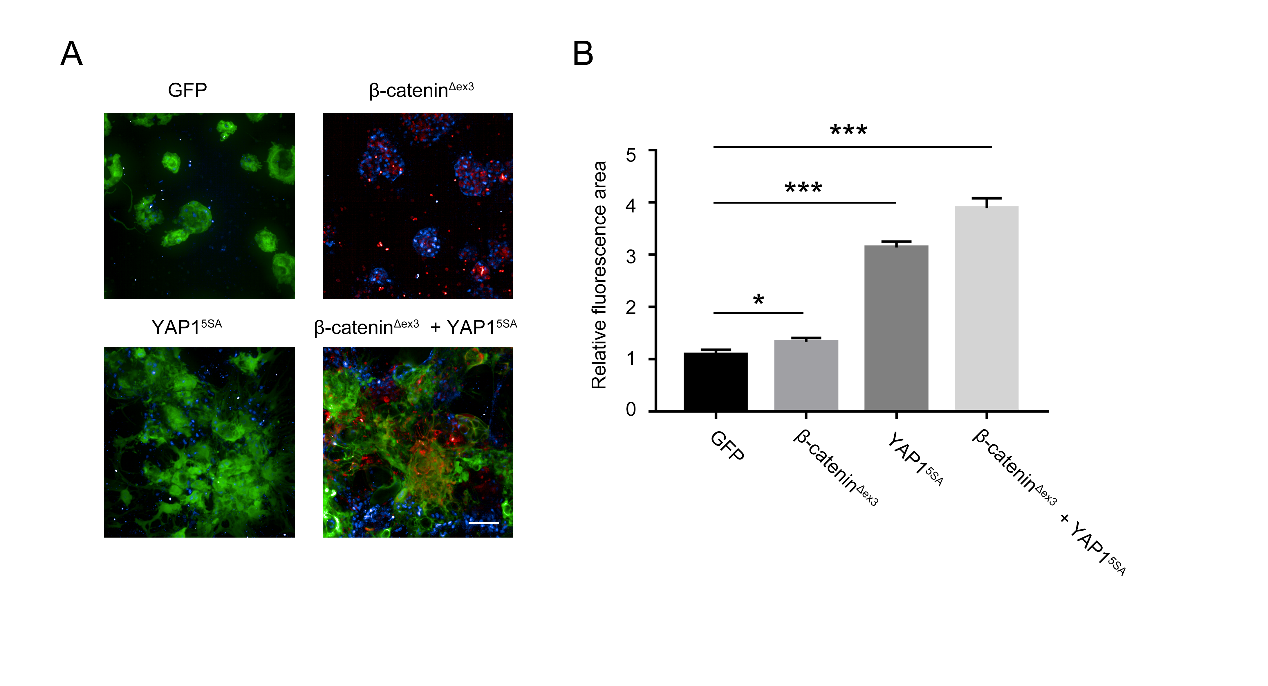


**Supplementary Figure 2 ∣ Human fetal liver organoids were transfected with lentiviral GFP, β-catenin^Δex3^-mCherry, YAP1^5SA^-GFP, or β-catenin^Δex3^-mCherry+YAP1^5SA^-GFP.** (A) Representative confocal images of transfected organoids. Scale bar, 100 μm. (B) Quantitative data were presented as means±SD (n=4).

**Supplementary Figure 3**


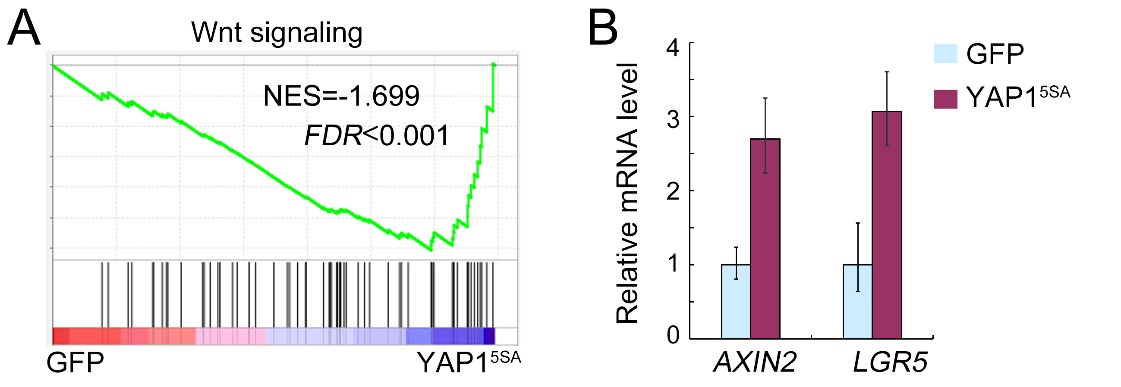


**Supplementary Figure 3 ∣ Wnt-β-catenin signaling was activated in YAP1-activated human fetal liver organoids.** (A) GSEA enrichment analysis of RNA sequencing data for the GFP and YAP1-transfected organoids. The genes of YAP1-activated organoids were enriched in Wnt signaling. FDR *q*<25%. NES, normalized enriched score. (B) qRT–PCR was performed to examine Wnt target genes in GFP and YAP1-transfected organoids. *H3* was used as an internal control. Data were presented as means±SD (n=3).

**Supplementary Figure 4**


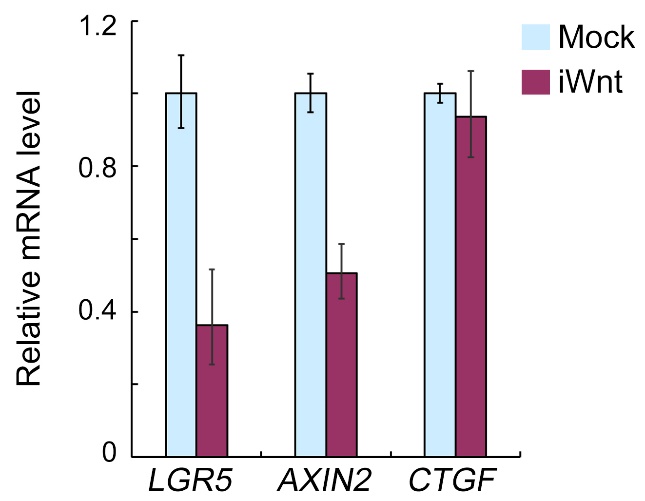


**Supplementary Figure 4 ∣ Abolishing transcriptional activity of β-catenin by IWP-2 treatment had no obvious effect on YAP1^5SA^-driven HB progression.** qRT–PCR was performed to examine Wnt target genes (*LGR5* and *AXIN2*) and YAP1 target gene (*CTGF*) in 5C-medium cultured YAP1-activated HB organoids. iWnt: Wnt inhibition by 5μM IWP-2 treatment. *H3* was used as an internal control. Data were presented as means±SD (n=3).

**Supplementary Figure 5**


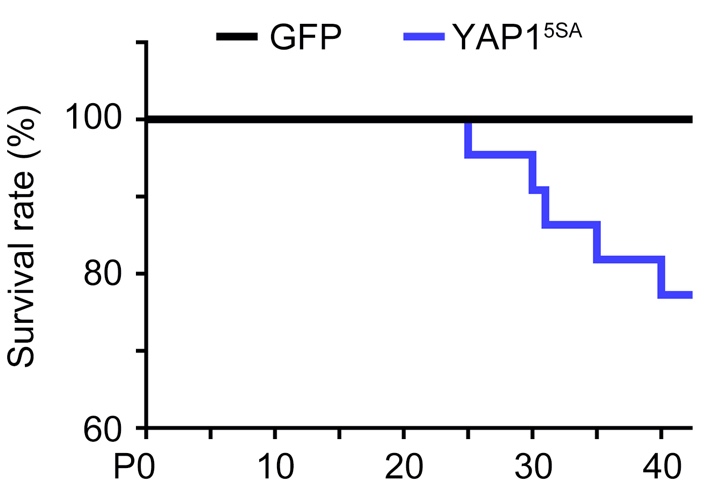


**Supplementary Figure 5 ∣ Kaplan–Meier survival curves of M-NSG mice transplanted with GFP**-**transfected and YAP1-activated HB organoids.** GFP and YAP1-transfected organoids (~2$\times$10^5^ cells) were injected into the liver capsule of M-NSG mice of aged 5–7 weeks. n=22 mice per group.

**Supplementary Figure 6**


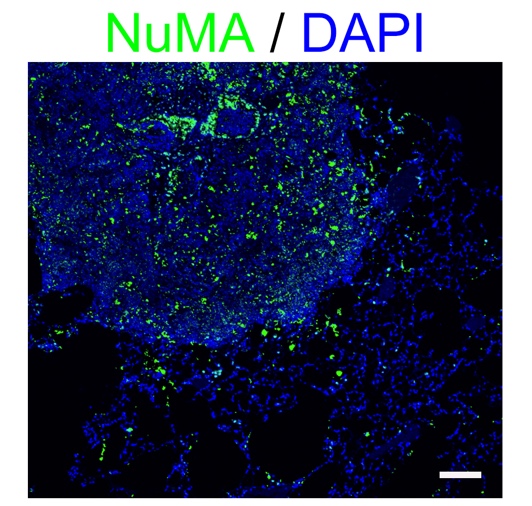


**Supplementary Figure 6. The lung metastatic foci originated from YAP1-activated human HB organoids.** Immunofluorescence analysis of human marker NuMA in metastatic foci in lung. Scale bar, 100 μm.

**Supplementary Figure 7**


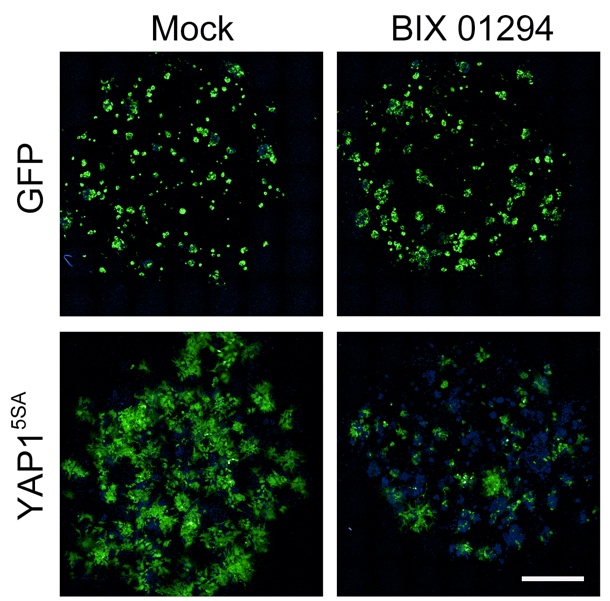


**Supplementary Figure 7. BIX 01294 inhibited the growth of YAP1-activated HB organoids for 7 days without affecting GFP-transfected organoids.** GFP and YAP1-transfected organoids at D14 were pretreated with BIX 01294 (200 nM) or Mock (DMSO) for 7 days in 5C-medium at 37°C before screening at D21. Hoechst was applied before screening. Scale bar, 1 mm. n=4.

**Supplementary Figure 8**

**
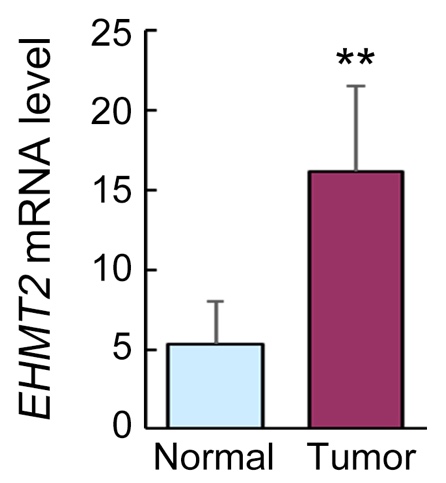
**

**Supplementary Figure 8. Data from public HB database suggested *EHMT2* was up-regulated in HB.** The data were obtained from the HBprem DataBase (http://www.hbpremdb.com/download.jsp). The *EHMT2* mRNA levels were compared between the tumors and normal tissues. Data were presented as means±SD (n=5).

**Supplementary Table 1. Gene expression profile in YAP1-activated human fetal liver organoids versus the mock (in spreadsheet)**

**Supplementary Table 2. Gene sets of “HB signature genes” and “HB down-regulated genes” (in spreadsheet)**

**Supplementary Table 3. *EHMT2* shRNA sequences**

| *EHMT2*-CDS-shF | CTCTTTCTCGAGAAAGAGCCATGAACTCTCTCGTTTTTGGAATTCTCGACCTCGAGACAA |
| --- | --- |
| *EHMT2*-CDS-shR | ATGGCTCTTTCTCGAGAAAGAGCCATGAACTCTCTCGCCGGTGTTTCGTCCTTTCCAC |
| *EHMT2*-3’UTR-shF | AGAGATCTCGAGATCTCTGGTCAGGAATGTGTGTTTTTGGAATTCTCGACCTCGAGACAA |
| *EHMT2*-3’UTR-shR | GACCAGAGATCTCGAGATCTCTGGTCAGGAATGTGTGCCGGTGTTTCGTCCTTTCCAC |

**Supplementary Table 4. Primers for qRT-PCR**

| Primers | Oligonucleotide sequence (5'-3') |
| --- | --- |
| h-*CTGF*-F | AAAAGTGCATCCGTACTCCCA |
| h-*CTGF*-R | CCGTCGGTACATACTCCACAG |
| h-*CYR61*-F | CTCGCCTTAGTCGTCACCC |
| h-*CYR61*-R | CGCCGAAGTTGCATTCCAG |
| h-*EHMT2*-F | TGACTGCGTGCTGTTATT |
| h-*EHMT2*-R | GCTTGCGGTTGAGTTG |
| h-*AXIN2*-F | AGTGTGAGGTCCACGGAAAC |
| h-*AXIN2*-R | CTTCACACTGCGATGCATTT |
| h-*LGR5*-F | TCCACTTTGCCATCCCTAA |
| h-*LGR5*-R | GGTCGTCCATACTGCTGTTG |
| h-*DKK1*-F | CCTTGAACTCGGTTCTCAATTCC |
| h-*DKK1*-R | CAATGGTCTGGTACTTATTCCCG |
| h-*COL2A1*-F | GGAAGAGTGGAGACTACTGGATTGAC |
| h-*COL2A1*-R | TCCATGTTGCAGAAAACCTTCA |
| h-*TNFRSF19*-F | GGCTGTTCCAGTCCAGTCTC |
| h-*TNFRSF19*-R | TGGATGACAGCACCACTCTC |
| h-*NPNT*-F | TGGCAAACTGTCAGTATGGCT |
| h-*NPNT*-R | CTTGCAGATGTAGCTCCCAAA |
| h-*MNTA3*-F | TCTCCCGGATAATCGACACTC |
| h-*MNTA3*-R | CAAGGGTGTGATTCGACCCA |
| h-*CST1*-F | GACACCTGTGCCTTCCATGA |
| h-*CST1*-R | CCTGGATTTCACCAGGGACC |
| h-*PCP4*-F | ATGAGTGAGCGACAAGGTGC |
| h-*PCP4*-R | ACTGAGACTGAATGGCCACC |
| h-*EDN3*-F | GGGACTGTGAAGAGACTGTGG |
| h-*EDN3*-R | AGACACACTCCTTGTCCTTGTA |
| h-*C9orf152*-F | TGACTCAGCAAGGAACCGGA |
| h-*C9orf152*-R | TTGTTTGGGTATTGCCCACCT |
| h-*PEG10*-F | GAGCACCAGGGATTTCTCAGT |
| h-*PEG10*-R | GGTAGTTGTGCATCAGGTAGTG |
| h-*OLR1*-F | TTGCCTGGGATTAGTAGTGACC |
| h-*OLR1*-R | GCTTGCTCTTGTGTTAGGAGGT |
